# Pulmonary cryptococcosis coexisting with adenocarcinoma: a case report and review of the literature

**DOI:** 10.1186/s13256-018-1853-2

**Published:** 2018-11-02

**Authors:** Liyang Li, Liang Zhuang, Jian Zhou, Changzhou Shao

**Affiliations:** 1 Shanghai Respiratory Research Institute, Department of Pulmonary Medicine, Zhongshan Hospital, Fudan University, Shanghai, 200032 China; 2 Cadre Ward, Sanming First Hospital, Sanming, 365000 Fujian Province China

**Keywords:** Pulmonary cryptococcosis, Adenocarcinoma, Pneumonia, Fungal infection, Abbreviations, CEA Carcinoembryonic antigen, CRP C-reactive protein, CT Computed tomography, PAS Periodic acid-Schiff, PCT Procalcitonin

## Abstract

**Background:**

Pulmonary cryptococcosis is a common fungal infection frequently seen in immunocompromised patients. Owing to its nonspecific clinical and radiographic features, the differential diagnosis with secondary tuberculosis, malignant tumor, and bacterial pneumonia is sometimes difficult. Many case reports have focused on misdiagnosis of pulmonary cryptococcosis as a malignant tumor. But to the best of our knowledge, the coexistence of pulmonary cryptococcosis and malignant tumor is rarely presented.

**Case presentation:**

A 52-year-old immunocompetent Han Chinese woman was presented to our emergency department complaining of headache and vomiting accompanied by postural changes. She was diagnosed with pulmonary cryptococcosis according to results of laboratory tests, computed tomography, and percutaneous lung biopsy. Owing to the poor therapeutic effects of 6-month fluconazole treatment, she underwent a second percutaneous lung biopsy and was diagnosed with pulmonary cryptococcosis coexisting with adenocarcinoma. Delayed treatment of malignant tumor resulted in lymph node metastasis, higher pathologic stage, and probably poorer prognosis.

**Conclusions:**

Our patient’s case serves as a reminder not to misdiagnose pulmonary cryptococcosis coexisting with adenocarcinoma.

## Background

Pulmonary cryptococcosis is a fungal infection due to the inhalation of *Cryptococcus neoformans* or *Cryptococcus gattii* spores [1]. It usually occurs in immunocompromised patients, especially in those with impaired T-cell-mediated immune deficiencies [2]. Deposition of spores in the alveoli may lead to latent infection in the lung or spread to the central nervous system through the bloodstream, depending on patients’ immune status [3]. The diagnosis of pulmonary cryptococcosis is challenging, given its nonspecific clinical and radiographic features. The differential diagnosis with secondary tuberculosis, malignant tumor, and bacterial pneumonia is sometimes hard. Many case reports have focused on misdiagnosis of pulmonary cryptococcosis as a malignant tumor [3–5]. But to the best of our knowledge, the coexistence of pulmonary cryptococcosis and malignant tumor was only presented in a few reports [6–10].

In this report, we describe a case of an immunocompetent woman who was first diagnosed with pulmonary cryptococcosis by percutaneous lung biopsy. But after 6-month antifungal therapy, a part of her lesions was not resolved, and then the second biopsy showed adenocarcinoma.

## Case presentation

**Fig. 1:**
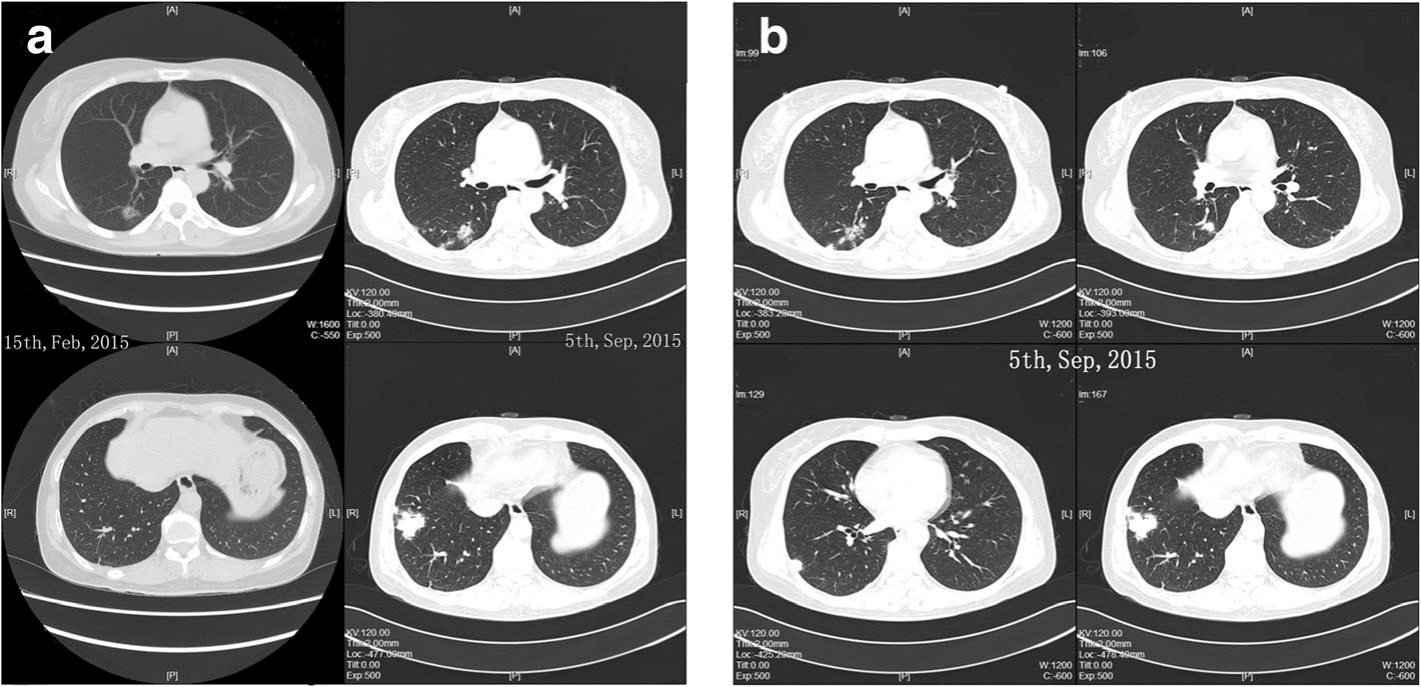
**a** The nodule in the right posterior segment appeared first, followed by the masses in the patient’s right lateral basal segment. **b** At the patient’s first admission to our hospital, computed tomography showed scattered multiple nodules and masses in her right lateral basal and posterior segments

A 52-year-old Han Chinese woman who worked as a teacher was presented to our emergency department complaining of headache and vomiting accompanied by postural changes. She had no respiratory symptoms and denied other discomfort. Computed tomography (CT) of her chest showed multiple nodules and masses in her right lower lung lobe (Fig. 1). Laboratory data, including results of routine blood tests and tumor markers (carcinoembryonic antigen [CEA] 4.1 ng/ml), were all normal. Finally, she was diagnosed with posterior circulation ischemia and received symptomatic treatment. She did not take the abnormalities in her lung seriously and declined to undergo further examination.

After nearly 7 months, the patient came to our respiratory outpatient department and underwent enhanced CT so that we could observe the changes in her lung, which showed scattered multiple nodules and masses in her right lateral basal and posterior segments, more serious than the previous time (Fig. 1). Hospitalization was recommended for further examination and treatment. She had cough as her only respiratory symptom and denied sputum, fever, chest pain, wheezing, malaise, weight loss, or other symptoms. She had not recently traveled or had contact with pigeons’ droppings or with soil, and she had no smoking or alcohol consumption history. Her family members included a healthy husband and a daughter. Her medical history included thyroid adenoma resection 13 years earlier. She had not taken any medicine before she was admitted to our hospital.

**Fig. 2:**
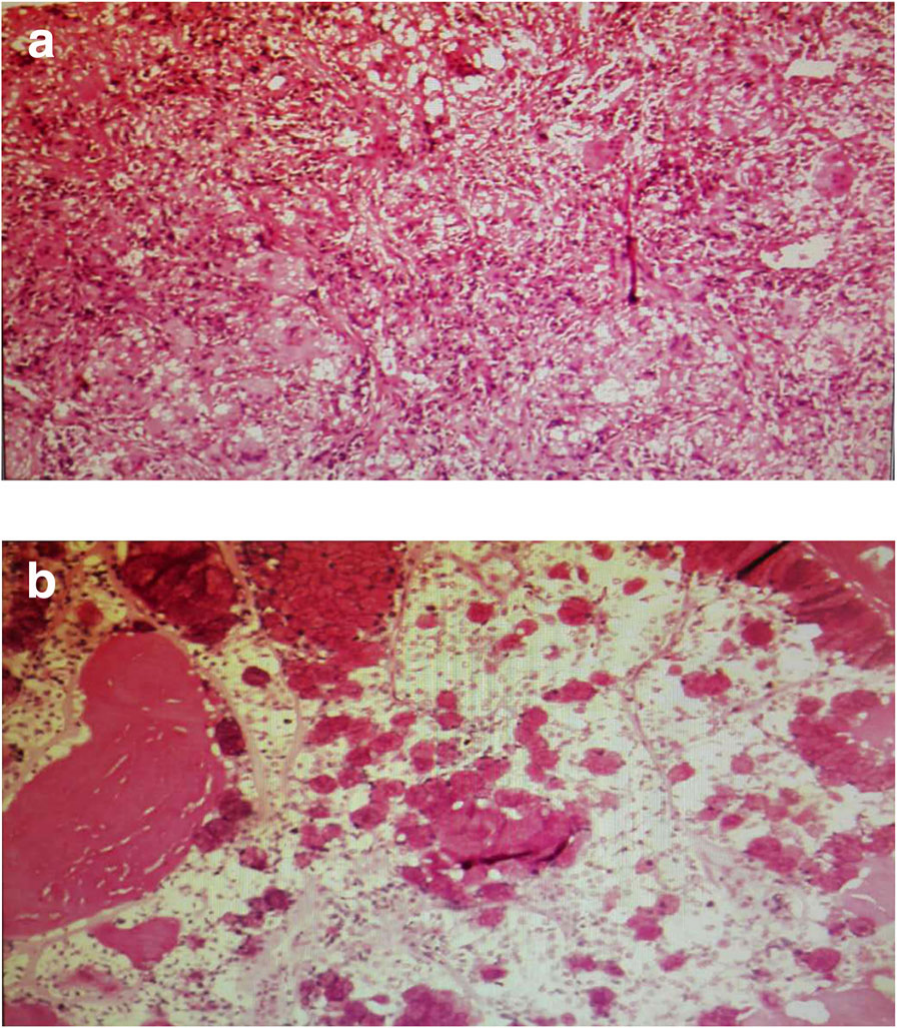
**a** Histopathological examination of percutaneous lung biopsy from the right posterior segment revealed granulomatous inflammation. **b** Periodic acid-Schiff stains the yeast wall a red color, suggesting pulmonary cryptococcosis

Physical examination revealed slightly decreased breath sounds at the right base upon auscultation. The result of the neurological examination was normal. On admission, her pulse was 106 beats/min, blood pressure 130/70 mmHg, and temperature 36.6 °C. Laboratory data, including results for blood cell count, platelet count, renal and liver function, C-reactive protein (CRP), procalcitonin, urinalysis, and stool routine and tumor markers, were all normal, except that CEA was 9.0 ng/ml, higher than the previous measurement. According to the patient’s CT results, we considered that she might have pulmonary bacterial infection and prescribed moxifloxacin and ceftizoxime as empirical treatment. After 2 weeks of antibiotic therapy, another CT examination was performed to evaluate the therapeutic effects. Unfortunately, the lesions shown on the previous CT studies had not resolved, and patchy consolidation was reported, suggesting that another diagnosis, such as pulmonary cryptococcosis, secondary pulmonary tuberculosis, or malignant tumor, should be taken into consideration. After that, a tuberculosis infection T-cell spot test (T-SPOT.*TB*; Oxford Immunotec, Marlborough, MA, USA) and tests for autoimmune antibodies were performed, but the results were all negative. A histopathological examination of percutaneous lung biopsy from the right posterior segment revealed granulomatous inflammation, and periodic acid-Schiff (PAS) staining showed red-colored yeast walls, suggesting pulmonary cryptococcosis (Fig. 2). The patient was then treated with fluconazole 0.2 g twice daily, and her condition was monitored with regular CT examinations.

**Fig. 3:**
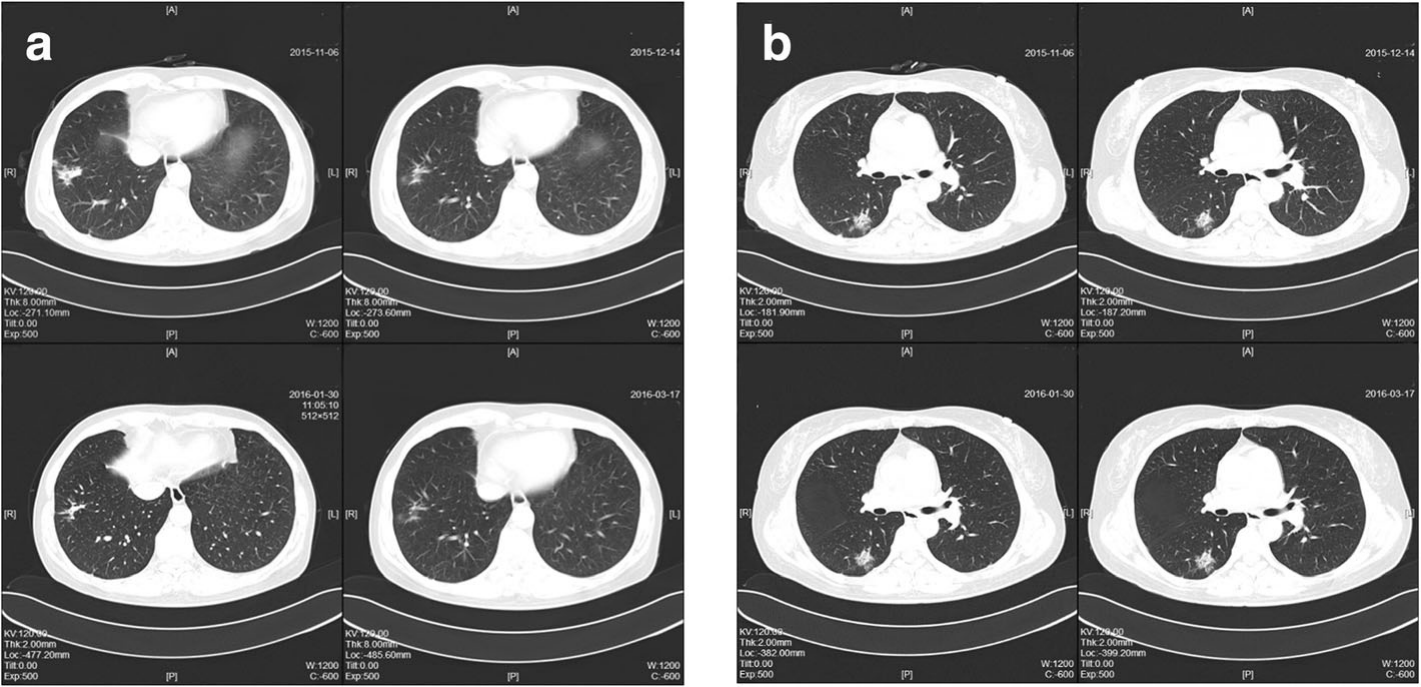
During her 6-month treatment, the patient underwent computed tomographic examination four times. **a** Great improvement in the patient’s lesion of the right lateral basal segment. **b** The nodule in the right posterior segment remained almost the same as before

During 6-month treatment with fluconazole, the patient underwent CT examination a total of four times, showing great improvement in her lesion of the right lateral basal segment, but the lesion in her posterior segment remained almost the same as before (Fig. 3). Two months after her drug withdrawal, she had surgery for a femoral neck fracture with cannulated screw internal fixation as an incidental event. CT performed as a routine preoperative examination showed that the multiple nodules and masses in her right posterior segment still had not resolved.

**Fig. 4:**
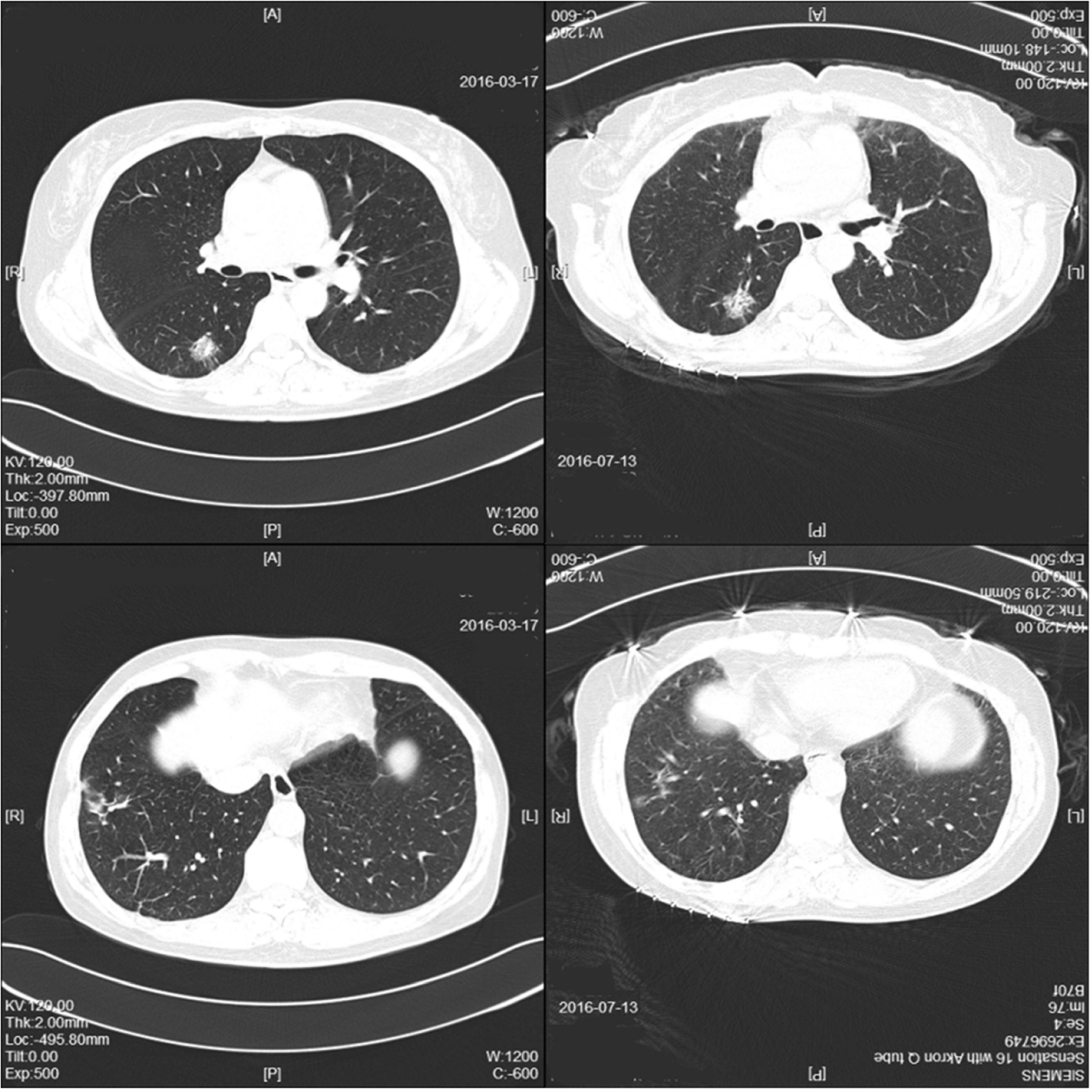
Two months after an operation using cannulated screw internal fixation for the patient’s femoral neck fracture, computed tomography showed that the size of the lesion in her right posterior segment had increased, though the lesion in her right lateral basal segment had decreased

**Fig. 5:**
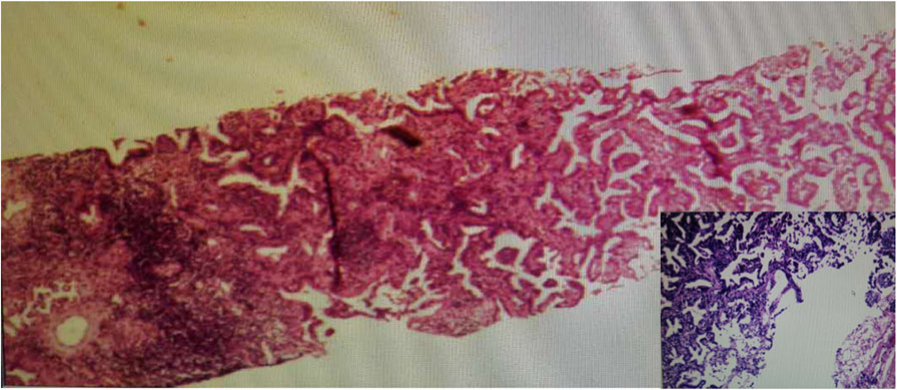
Histopathological examination of percutaneous lung biopsy from the nodule in the right posterior segment was performed again, showing adenocarcinoma

Another 2 months after the operation, she was admitted to our respiratory department for further examination. An enhanced CT study showed that the lesion in her right posterior segment had increased, though the lesion in her right lateral basal segment was decreased (Fig. 4). She denied any clinical symptoms, and her laboratory test results were normal. This extraordinary phenomenon led us to think about another diagnosis in this case. Another histopathological examination of percutaneous lung biopsy from the nodule in the right posterior segment was performed. Finally, the result, as we suspected, was adenocarcinoma (Fig. 5).

Two days later, the patient turned to our department of thoracic surgery. Thoracoscopy-assisted radical surgery of the right lower lobe was performed. Postoperative pathology showed the adenocarcinoma in her right lower lobe and metastasis in groups 2, 4, and 9 lymph nodes, suggesting stage IIIA (pT1bN2M0) cancer. Four cycles of chemotherapy (pemetrexed 800 mg and carboplatin 500 mg) were administered. Illness evaluation was stable disease. In consideration of the metastasis in the mediastinal lymph nodes, the patient received radiation treatment (50 Gy in 25 fractions) after chemotherapy. The patient’s CEA reduced to 1.12 ng/ml after that. She was regularly followed until January 2018, when a CT examination showed multiple nodules in her right middle lobe. She then received gefitinib 250 mg daily, and the nodules had disappeared in May 2018 when she came to a visit for disease assessment. Gefitinib has been continued.

## Discussion and conclusions

Our patient was finally diagnosed with pulmonary cryptococcosis coexisting with adenocarcinoma. She was immunocompetent without any history of contact with poultry or pollution. The diagnosis was established by two percutaneous lung biopsies. The first time, the pathological report was granulomatous inflammation, and PAS staining was positive, suggesting pulmonary cryptococcosis. Fluconazole was used for 6 months, and the lesions partly resolved. The second biopsy of another lesion was performed 10 months after pulmonary cryptococcosis was diagnosed, revealing adenocarcinoma. Postoperative pathology showed metastasis in lymph nodes, suggesting stage IIIA (pT1bN2M0) cancer. Compared with the cases reported in the literature, our patient’s definitive diagnosis took more time. Multiple nodules with different pathogeny and the shrinkage of the lesion were obstacles to get the correct diagnosis. Delayed treatment of malignant tumor resulted in higher pathologic stage and poorer prognosis.

Cryptococcosis used to be studied in patients with acquired immunodeficiency syndrome in many cases. Currently, it is becoming an important infection in human immunodeficiency virus-negative patients [11], particularly in those with significant underlying immunosuppression such as organ transplant, long-term glucocorticoid treatment, hematologic malignancies, diabetes mellitus, hepatic cirrhosis, among others [12, 13]. In China, pulmonary cryptococcosis ranked as the third most common pulmonary fungal infection, and most Chinese patients with pulmonary cryptococcosis do not have underlying diseases [14]. Our patient was an immunocompetent patient without an immunity-associated medical history.

The pathogen of pulmonary cryptococcosis is the following two subspecies of the *Cryptococcus* family: *C. neoformans* and *C. gattii*. They are abundant in pigeon droppings, contaminated soil, and barns. *C. neoformans* majorly affects immunocompromised patients worldwide, whereas *C. gattii* is usually related to immunocompetent cryptococcosis in tropical and subtropical areas [12, 15]. The patient denied any history of contact with birds or polluted environment and had not recently traveled. We had no idea about when and where she was incidentally infected by spores.

The common symptoms of pulmonary cryptococcosis are cough, fever, pleuritic pain, dyspnea, hemoptysis, fatigue, and weight loss [16], all of which are symptoms overlapping the clinical features of lung cancer. Immunocompetent patients with pulmonary cryptococcosis usually have cough as their only symptom, and one-fourth remain asymptomatic [11], which makes it difficult to make the diagnosis unequivocally. Our patient had no respiratory symptoms except a little cough for a short time. We decided that a broad range of differential diagnoses should be considered according to her clinical symptoms.

The radiographic features of pulmonary cryptococcosis are also atypical. The common CT manifestations of pulmonary cryptococcosis were solitary or multiple pulmonary nodules or masses [17], making it difficult to distinguish cryptococcosis from malignant tumor and bacterial pneumonia. It has been reported that among cases of pulmonary cryptococcosis coexisting with lung cancer, adenocarcinoma is the main histological type, probably because cryptococcosis is apt to occur in the periphery of the lung, where adenocarcinoma is commonly found [18]. Cavitation and halo sign are more frequently observed in immunocompromised patients [19]. Pleural effusion and lymphadenectasis sometimes occur as well [20]. Many case reports describe that pulmonary cryptococcosis mimicked lung cancer [3–5, 21]. In our patient’s case, however, pulmonary cryptococcosis was coexistent with adenocarcinoma, which is rarely reported. On the basis of her CT examination, we can find that the nodule in her right posterior segment appeared first, followed by the masses in her right lateral basal segment. After antifungal therapy, her masses in her right lateral basal segment resolved greatly compared with the nodule in the right posterior segment, which was finally diagnosed as adenocarcinoma (Fig. 1a). We supposed that adenocarcinoma occurred earlier than pulmonary cryptococcosis. It was reported that malignant tumor might lead to immunodeficiency and result in cryptococcosis [9].

Laboratory tests are helpful for diagnosing respiratory diseases. But in pulmonary cryptococcosis, laboratory tests such as white blood count, CRP, and renal and liver function, are usually normal. We believe that tumor markers may be the main points of interest in laboratory tests to identify pulmonary cryptococcosis and malignant tumor. Serum cryptococcal antigen titer is often used to diagnose cryptococcosis [1]. Because there was no serum cryptococcal antigen titer available in our hospital, we did not detect cryptococcal antigen. Identification and culture of organisms in patients’ sputum or bronchoalveolar lavage fluid is also a common method to make a definitive diagnosis. However, fungus culture usually produces false-negative results [13, 22]. It has been reported [2, 16] that different subspecies of *Cryptococcus* may cause different immune responses and require different therapy. But the clinical culture does not classify the subspecies as routine. Our patient’s CEA was normal (the normal value is below 15 ng/ml in our hospital) without any abnormalities in other laboratory data. The increase of the patient’s CEA from 4.1 ng/ml to 9.0 ng/ml in 7 months may have prompted us to be aware of the possibility of lung adenocarcinoma. The patient could not produce sputum, so culture of the sputum was abandoned. In order to gain a definite pathological diagnosis, percutaneous lung biopsy was carried out. Histopathological and immunohistochemical studies showed granulomatous inflammation with positive PAS staining, consistent with the classic manifestation of pulmonary cryptococcosis [2]. Taking pathological results as a gold standard, owing to the regression of one of the lesions after antifungal therapy, we excluded the diagnosis of malignant tumor, which resulted in a missed diagnosis.

Regarding the treatment of cryptococcosis, clinical guidelines recommend oral fluconazole for patients with mild to moderate symptoms and amphotericin B plus flucytosine followed by fluconazole for severe symptoms or disseminated cryptococcosis [23]. A recent study proposed that surgical resection of pulmonary cryptococcoma in patients with cryptococcal meningitis may improve the prognosis [24]. Also, another group came to the conclusion that pulmonary cryptococcosis resolves in most patients with or without specific antifungal therapy [25]. The treatment of pulmonary cryptococcosis is totally different from that of adenocarcinoma. Misdiagnosis could lead to delayed treatment, resulting in cancer progression or disseminated infection.

Our patient’s case serves as a reminder of the possibility of dualism in making a diagnosis of pulmonary multiple nodules on the basis of CT examination. It reminded us of the possibility of pulmonary cryptococcosis coexisting with adenocarcinoma, even though pathology showed Cryptococcus infection and antifungal therapy was partly effective. Regular computed tomographic examination is necessary, and tumor markers should be followed until the lesions resolve completely. If some of the nodules remain unchanged after treatment or even become larger, malignant tumor, especially adenocarcinoma, should be taken into consideration. Pathological results of one lesion cannot represent the property of all the lesions. Close follow-up and biopsy of multiple lesions may be necessary in such cases.
